# Anatomic Peculiarities Associated with Axial Elongation of the Myopic Eye

**DOI:** 10.3390/jcm12041317

**Published:** 2023-02-07

**Authors:** Jost B. Jonas, Mukharram M. Bikbov, Ya-Xing Wang, Rahul A. Jonas, Songhomitra Panda-Jonas

**Affiliations:** 1 Department of Ophthalmology, Medical Faculty Mannheim of the Ruprecht-Karis-University, 68167 Mannheim, Germany; 2 Institute for Clinical and Scientific Ophthalmology and Acupuncture Jonas & Panda, 69120 Heidelberg, Germany; 3 Privatpraxis Prof. Jonas und Dr. Panda-Jonas, 69115 Heidelberg, Germany; 4 Ufa Eye Research Institute, Ufa 450008, Russia; 5 Beijing Institute of Ophthalmology, Beijing Tongren Hospital, Capital Medical University, Beijing Ophthalmology and Visual Sciences Key Laboratory, Beijing 100041, China; 6 Department of Ophthalmology, University of Cologne, 50923 Cologne, Germany

**Keywords:** myopia, axial elongation, Bruch´s membrane, retinal pigment epithelium, macula, choroid, retina

## Abstract

Purpose: To describe anatomical peculiarities associated with axial elongation in the human myopic eye. Methods: Reviewing the results of previous histomorphometrical investigations of enucleated human globes, as well as reviewing findings obtained in population-based studies and hospital-based clinical investigations of myopic patients and non-myopic individuals. Results: Myopic axial elongation is associated with a change from a mostly spherical eye shape to a prolate ellipsoid form. It is combined with choroidal and scleral thinning, most pronounced at the posterior pole and less pronounced in the fundus midperiphery. In the fundus midperiphery, the retina and density of the retinal pigment epithelium (RPE) and photoreceptors decrease with a longer axial length, while in the macular region, retinal thickness, RPE cell density, and choriocapillaris thickness are not related to axial length. With axial elongation, a parapapillary gamma zone develops, leading to an enlargement of the optic disc-fovea distance and a decrease in angle kappa. Axial elongation is also correlated with an increase in the surface and volume of Bruch’s membrane (BM), while BM thickness remains unchanged. Axial elongation causes moderately myopic eyes to show a shift of BM opening to the foveal direction so that the horizontal disc diameter becomes shorter (with a consequent vertical ovalization of the optic disc shape), a temporal gamma zone develops, and the optic nerve exit takes an oblique course. Features of high myopia are an enlargement of the RPE opening (myopic parapapillary beta zone) and BM opening (secondary macrodisc), elongation and thinning of the lamina cribrosa, peripapillary scleral flange (parapapillary delta zone) and peripapillary choroidal border tissue, secondary BM defects in the macular region, myopic maculoschisis, macular neovascularization, and cobblestones in the fundus periphery. Conclusions: These features combined may be explained by a growth in BM in the fundus midperiphery leading to axial elongation.

## 1. Introduction

Axial myopia, as one of the most common causes of irreversible vision impairment and blindness, is associated with a wide variety of anatomical characteristics [1,2,3,4,5,6,7,8]. It is characterized by an enlargement elongation of the anterior–posterior globe diameter. From birth onwards, the globe mostly actively enlarges in all directions, resulting in a spherical shape of the eye. The sagittal diameter elongates in that period of the first two to three years of life from 17 mm at birth to approximately 21 to 22 mm. At the end of the second to third year of life, the lens and cornea have reached mostly adult dimensions and do not change much after that period. The sagittal eye diameter then elongates to adapt the length of the optical axis to the requirements given by the optical properties of the cornea and lens, with the aim to have the image of an object at distance focused exactly on the photoreceptor outer segments in the foveola. This so-called process of emmetropization results in axial myopia; the optical axis becomes longer than needed for emmetropia. The mechanism underlying the process of emmetropization and myopization remains unclear. Knowledge of its detail may be instrumental to better understand the pathogenesis of high myopia and pathologic myopia and to develop therapeutic strategies against myopia-related vision impairment and blindness. Therefore, in this review, we summarized findings obtained in previous and recent histologic and clinical studies to put together a puzzle consisting of numerous single morphological observations. Composing such a picture may lead to a better understanding of the process of myopic axial elongation, may be useful to correlate anatomical findings with clinical psychophysical parameters such as visual acuity and visual field, and may be helpful to elucidate the pathomechanism of pathological myopia including myopic maculopathy and high-myopia-related optic neuropathy.

## 2. Methods

This review is based on the results of histomorphometric studies including human globes that had been enucleated for reasons such as malignant uveal melanomas or painful secondary angle-closure glaucoma. The review also summarizes observations made in population-based studies and hospital-based clinical investigations of myopic patients and non-myopic individuals. According to the guidelines laid down in the World Medical Association Declaration of Helsinki, the Medical Ethics Committee II of the Medical Faculty Mannheim of the Heidelberg University approved the histological studies. It waived the necessity of an informed written consent signed by the study participants, as the enucleations had taken place about 25 to 60 years before the investigations were carried out. Out of a pool of slides of more than 200 globes, the histomorphometric studies examined, at various ocular locations, the ora serrata region; the equator; the midpoint between the equator and the posterior pole, and at the posterior pole; morphometric parameters such as the thickness of Bruch´s membrane (BM), sclera, and outer retinal nuclear layer and inner nuclear layer; the density of the retinal pigment epithelium (RPE) cells and photoreceptors; the thickness and density of the choriocapillaris; the total retinal thickness; the size of the parapapillary alpha zone, beta zone, gamma zone, and delta zone; and the length of the longitudinal part of the ciliary muscle (Brücke´s muscle) [5]. In clinical cross-sectional and longitudinal studies, we assessed characteristics of the myopic fundus including the size and shape of the parapapillary zones, the optic disc–fovea distance, the angle kappa between the temporal superior arterial arcade and the temporal inferior arterial arcade, the distance between the temporal superior arterial arcade and temporal inferior superior arterial arcade on a vertical line running through the fovea, the size and shape of the optic disc and BM opening, and the shape of the optic nerve exit through the optic nerve canal. In longitudinal studies, we assessed changes in these morphological characteristics of the myopic fundus over a period of 10 years and more. The studies including patients were based on a population-based recruitment and hospital-based recruitment of the study participants. The observations made in these studies were supplemented by and compared with findings obtained in previous investigations by other researchers.

## 3. Results

### 3.1. Sclera

With an increasing axial length, the sclera thickness decreases—most markedly at the posterior pole and least markedly at the ora serrata or anterior to it [9,10,11,12,13,14,15,16]. In individuals aged 3+ years, the cross-section area and the volume of the sclera are independent of age and of axial length [17,18]. This suggests that, up to the third year of life, the sclera may actively grow in the framework of the general enlargement of the eye in that period. It also indicates that the axial-elongation-related thinning of the sclera after the third year of life is mostly a re-arrangement of the available scleral tissue and does not indicate growth of the sclera [10,12,19]. It points against a primary role the sclera may play in the process of axial elongation and its regulation. Scleral thickness data obtained at various locations in the anterior ocular segment are associated with each other; however, they are not correlated with data on sclera thickness measured in the posterior half of the eye, and vice versa [15]. Scleral thickness independent of the location is not related to the central or peripheral corneal thickness, independently of axial length [20]. The finding that a longer axial length correlates with a thinner sclera posterior to, but not anterior to, the ora serrata suggests that the myopic axial elongation takes place predominantly in the posterior segment.

### 3.2. Scleral Staphyloma

Scleral staphylomas are a feature of highly myopic eyes. They have been described as outpouchings of a circumscribed region of the posterior sclera [21,22,23,24,25,26,27,28,29,30,31,32,33,34,35,36,37,38]. Scleral staphylomas show a relatively abrupt scleral thinning at the staphyloma edge; a pronounced de-arrangement of scleral collagen fibrils; a marked, segmentally accentuated, choroidal thinning, most marked at the staphyloma border; and spatially correlated BM defects. The thickness and density of the choriocapillaris and RPE cell layer and BM thickness do not differ significantly between the staphylomatous versus non-staphylomatous regions [39,40]. Usually occurring in highly myopic eyes, scleral staphylomas can also be found in non-highly myopic eyes, such as those with retinitis pigmentosa or with localized Bruch’s membrane (BM) defects [40,41].

### 3.3. Choroid

Choroidal thickness decreases with axial elongation, most markedly in the subfoveal region and least markedly in the equatorial region [42,43,44,45,46,47,48,49]. Myopic choroidal thinning affects predominantly Haller´s layer and Sattler’s layer, while the choriocapillaris (at the posterior pole) is least or not at all affected [50,51,52]. According to some studies, the density and thickness of the choriocapillaris at the posterior pole was not related to the axial length in eyes without pathological myopia [51,52]. This fits with the finding that BM thickness and length at the posterior pole were not related to axial length [53,54].

### 3.4. Bruch’s Membrane

The thickness of BM, measured at any location of the eye, is not related to axial length, thus BM thickness in a highly myopic eye is similar to BM thickness in an emmetropic eye [53,55,56]. As the surface area of BM increases with a longer axial length, the volume of BM increases as well. This points to an active growth of BM with myopic elongation [57]. The findings obtained in human eyes were also found in enucleated eyes of guinea pigs [56].

Taking the association between the regional photoreceptor density and axial length or the regional RPE cell density and axial length as a surrogate for a segmental axial-elongation-related enlargement of BM, studies suggested that axial elongation is associated with, or due to, an enlargement (or growth) of BM predominantly in the midperiphery of the fundus [56,58]. This fits with the change in the eye shape roughly from a sphere in emmetropia to a prolate ellipsoid in axially elongated eyes [59,60,61,62,63,64,65,66].

The biomechanical properties of BM have recently been addressed [67,68,69]. Burst examinations revealed that BM could withstand a mean intraocular pressure (IOP) of 82 mmHg before rupturing. These and other findings suggested that BM is a nonlinear soft tissue (stiffer with stretch), and that the elastic modulus of BM is comparable to or higher than that of the sclera or any other ocular tissue [69,70,71,72,73,74,75,76,77]. This biomechanical strength of BM supports the notion of BM playing a biomechanically important role in general and for myopic axial elongation in particular.

### 3.5. Bruch’s Membrane Defects

Axial elongation beyond an axial length of approximately 26.0 or 26.5 mm is associated primarily with an enlargement of BM opening of the optic nerve head, followed by the development of secondary BM defects in the macular region [78,79,80,81,82]. These secondary BM defects correspond to patchy atrophies at stage 3 of myopic maculopathy [83]. They are associated with a complete loss of the RPE and choriocapillaris; with an almost complete absence of mid-sized and large choroidal vessels; and with a regionally more marked scleral thinning in the region of the defect, with a defect in the overlying RPE layer (usually larger than the BM defect), a defect in the overlying outer nuclear layer (usually slightly larger than the BM defect), a defect in the overlying inner nuclear layer (usually smaller than the BM defect), and so-called inner limiting membrane bridges. Within the BM defects, the inner scleral surface has direct contact with the inner retinal layers. A higher number and larger size of macular BM defects correlate with a longer axial length and a higher prevalence of scleral staphylomas. Examined at the border BM defects, the thickness of the choriocapillaris and BM and the RPE cell density are not significantly different from measurements obtained at other corresponding locations. Owing to the absence of RPE cells and photoreceptors, the macular BM defects are associated with a localized blind scotoma.

### 3.6. Retinal Pigment Epithelium (RPE)

The RPE cell density, measured in the midperiphery of the fundus, decreases with a longer axial length, while it is mostly independent of axial length in the macular region [56,58]. This fits with the notion that axial elongation occurs predominantly through an enlargement of the eye wall (i.e., BM) in the fundus midperiphery. It also agrees with the findings that the thickness and length of BM in the macular region are independent of the axial length [53,54].

The RPE opening of the optic nerve head enlarges with a longer axial length. This leads to the myopic parapapillary beta zone, defined as the parapapillary region with BM uncovered by RPE. It has to be differentiated from the glaucoma beta zone [84]. It is likely that, after the development and enlargement of a myopic beta zone, secondary defects in the RPE layer in the macular region develop, leading to “patchy atrophies” as a feature of stage 3 of myopic maculopathy.

### 3.7. Retinal Photoreceptor Layer

Assuming that the photoreceptors do not show a mitotic activity during childhood and later on, the density of the photoreceptors is supposed to decrease in myopic eyes because of the axial-elongation-related increase in the inner surface of the eye, namely an enlargement of the inner surface of BM. Histological studies have suggested that such an axial-length-related decrease in the photoreceptor density takes place predominantly in the fundus midperiphery and periphery of the fundus, while the density in the macular is less, or not at all, affected by axial elongation in eyes with non-pathological myopia. Correspondingly, the thickness of the retina decreased with a longer axial length in the fundus midperiphery, while the retinal thickness in the macular region was only slightly, or not at all, dependent on the axial length [85,86]. These findings support the notion that axial elongation is associated with an enlargement of the wall mainly in the fundus midperiphery [57].

### 3.8. Retina

#### 3.8.1. Retinal Length

Myopic axial elongation is associated with an increase in the distance from the posterior pole to the ora serrata, and to a minor degree with an elongation of the distance between the ora serrata and the scleral spur [87]. The diameter of the cornea is mostly independent of the axial length in eyes with primary axial myopia. This is in contrast to eyes with secondary myopia due to congenital glaucoma, in which the lengths of all parts of the eye wall including the cornea increase. The increased length of the ora serrata–posterior pole distance leads to a lengthening and stretching of the retina, namely its structures that connect the photoreceptors to the optic disc. These structures are the limiting membrane and the axons of the retinal ganglion cells or retinal nerve fibers. Stretching of the retinal nerve fibers may lead to their damage and loss, resulting in a non-glaucomatous optic nerve damage [88]. Stretching of the inner limiting membrane, in particular in the region of posterior scleral staphylomas, may contribute or lead to a maculoschisis. The myopic enlargement of the retinal underground, i.e., BM, will lead to a spreading and lower density of the retinal photoreceptors, resulting in a reduction in spatial resolution. Another factor causing a decreased spatial resolution will may be the decreased density of the cells of the retinal image processing system, namely the retinal ganglion cells, so that the size of a receptive field will grow with a longer axial length [89].

#### 3.8.2. Optic Disc–Fovea Distance

In the course of the myopic eye enlargement, the optic disc–fovea distance elongates [90]. This elongation is almost entirely due to the development and enlargement of the parapapillary, a BM-free gamma zone, while the length of BM, measured between the end of the gamma zone and the foveola, is independent of the axial length. The elongation of the disc–fovea distance leads to a lengthening of the inner limiting membrane and of the retinal nerve fibers in the papillo-macular bundle region. The retinal nerve fibers in the arcuate region can compensate for the increased distance to the optic disc by straightening their course to the optic nerve head [91]. It may be discussed that the lengthening and potentially stretching of the papillo-macular retinal nerve fibers may lead to damage and loss, resulting in a non-glaucomatous optic neuropathy with central scotomas, which cannot be explained by deep retinal changes in the macular region [88]. As also pointed out above, the stretching of the non-elastic inner limiting membrane may lead to the detachment of the deep retinal layers, with the inner retinal layers attached to the inner limiting membrane. An intraretinal schisis, i.e., maculoschisis, may develop.

#### 3.8.3. Cobblestones

“Cobblestones” or retinal cobble stone degenerations are found in the equatorial region of highly myopic eyes [92,93]. In histologic sections, the cobblestones are characterized by a thinned, although intact, BM; reduced choriocapillaris thickness and density; firm adhesion between a disorganized (and thinned) retina and BM; an absence of RPE within the cobblestone region except for a few islands of RPE cells; an absence of intraretinal or subretinal RPE proliferations; and a scleral thickness that did not differ significantly from other corresponding regions [93]. While the etiology of cobblestones in highly myopic eyes has remained unclear so far, it has been discussed that the myopia-associated enlargement of the inner ocular surface, namely the BM surface, leads to an increased strain in the RPE layer in the fundus periphery as well. It may lead to RPE layer defects in the shape cobblestones. Such a mechanism could explain the absence of RPE cells and the absence of an inner BM layer in the cobblestone region. In that model, Haller’s and Sattler’s layer of the choroid and the sclera would primarily be unaffected. This could be an example of a discrepancy between the local sequels of the myopic stretching between the RPE layer and the other layers of the ocular coat. One may discuss whether the etiology of the cobblestones located in the fundus periphery has similarities with the pathomechanism of the RPE layer in the macular region in the form of patchy atrophies.

#### 3.8.4. Axial-Elongation-Related Shift of the Different Ocular Layers

As the retina, RPE layer, BM, choriocapillaris, and Haller’s and Sattler’s layer of the choroid and the sclera are not firmly attached to each other, they may shift in a spatial relationship to each other [94,95,96,97]. Examples also discussed above consider the postulated shift of the RPE layer in the form of an enlargement of the RPE opening of the optic nerve head canal, leading to a myopic parapapillary beta zone, as well as the shift between the outer retinal layers away from the optic disc (in association with a sliding BM in association with the development of a parapapillary gamma zone) and the inner retinal layers adherent to the inner limiting membrane fixed at the optic disc border and potentially leading to a myopic maculoschisis. Other examples may be a shift of large choroidal vessels in relation to retinal vessels, occurring most often in the disc–fovea line and occurring correspondingly to the gamma zone enlargement in eyes with a parapapillary gamma zone without choroidal vessels. Interestingly, eyes with a gamma zone containing large choroidal vessels showed a choroidal vessel shift that was smaller than the increase in the gamma zone. Another example of ocular layers shifting in spatial relation to each other during myopic axial elongation may be the observation that the choroidal inter-vessel distance increased in the regions of an enlarging diffuse chorioretinal atrophy (stage II of myopic maculopathy) or in the regions of macular patchy atrophies. Interestingly, in some eyes with macular BM defects with choroidal vessels at their bottom, the inter-vessel distance in the choroidal large vessel layer did not markedly change [94,95,96,97].

The relative shift of the large choroidal vessels in relation to retinal landmarks may be partially due to a temporal shift of the BM opening in association with an enlargement of the parapapillary gamma zone. The constancy of the position of the large choroidal vessels in regions of macular BM defects may be due to a relative movement of the choriocapillaris, strongly adherent to the shifting BM in the framework of the developing BM defect, while the large choroidal vessels only loosely connected to the choriocapillaris may partially remain in the center of the BM defect.

### 3.9. Optic Nerve Head

#### 3.9.1. Optic Nerve Head Canal

The optic nerve head includes the optic nerve head canal with its layers of the lamina cribrosa as the opening in the peripapillary scleral flange, anteriorly followed by the choroidal opening (bordered by the peripapillary border of the choroid); the BM opening; the opening in the RPE layer; and the openings in the outer nuclear layer, inner layer, and retinal ganglion cell layer [84,98]. The retinal nerve fiber layer continues through the retinal nerve fibers into the intrapapillary compartment of the optic nerve head canal, while the inner limiting membrane may potentially continue across the optic nerve canal.

At birth, all layers of the optic nerve canal may be aligned to each other, resulting in an almost circular optic disc shape with a nerve fiber exit in an almost perpendicular angle. With increasing myopic axial elongation, the BMO shifts usually to the temporal direction. It leads to an overhanging of BM into the nasal part of the intrapapillary compartment and, correspondingly, to an absence of BM at the temporal disc border [99]. It leads to the development of a parapapillary gamma zone, defined as the parapapillary region without BM [100,101,102,103,104,105,106,107,108,109,110,111,112,113]. It occurs primarily without an enlargement of the BM opening. The reason for the shift of the BM opening to the macular region may be an enlargement (growth) of BM in the fundus midperiphery, as already discussed above [57]. The BM opening shift leads to an oblique exit canal for the retinal nerve fibers, with the macular fibers becoming posterior and leaving the eye through the oblique optic nerve canal in the anterior direction, before turning back through the optic nerve and heading for the nasal upper part of the orbit.

The BM opening shift with the overhanging of BM into the intrapapillary compartment leads to a reduction in the size of the optic disc, if the latter is defined as the ophthalmoscopically visible part of the lamina cribrosa, covered or uncovered by neuroretinal rim [96]. As the BM opening shift usually occurs in the temporal to temporal-inferior direction towards the fovea (which is located slightly inferior to the horizontal optic disc axis), the horizontal optic disc diameter shortens. This leads to a decrease in the optic disc size and to a change in the disc shape from a circular or slightly vertically oval form to a markedly vertically oval shape [96].

So-called “tilted discs” are optic discs in which BM is overhanging into the superior part of the intrapapillary compartment, with a corresponding absence of BM in the inferior parapapillary region (i.e., an inferior gamma zone). Owing to the vertical shift of the BM opening in the inferior direction, the vertical diameter of the optic disc is shortened while the horizontal disc diameter remains untouched [104,114,115,116,117,118]. This results in a horizontally oval disc shape, with an abundance of retinal tissue at the superior disc pole and only a thin retinal tissue at the inferior disc pole. In contrast to the term “tilted disc”, such an optic disc is not tilted but simply shows a BM opening shift in an unusual direction—here, in the inferior direction.

In eyes with a so-called “inversion situs papillae” or “inversion of the optic disc” with a primary exit of the retinal vessels into the nasal direction (before the vessel turns in the temporal direction), BM is overhanging into the temporal part of the intrapapillary compartment, with a corresponding absence of BM in the nasal parapapillary region (i.e., nasal gamma zone). It leads to a shortening of the horizontal disc diameter with a vertical ovalization of the optic disc shape (similar to eyes with a temporal shift of the BM opening) and a corresponding reduction in the optic disc size [119].

#### 3.9.2. Lamina Cribrosa

The lamina cribrosa forms the bottom of the optic nerve head and can be considered to be the cover of the opening of the peripapillary scleral flange. The thickness and size of the lamina cribrosa are mostly independent of the axial length in non-highly myopic eyes, while they enlarge in highly myopic eyes with a longer axial length [120,121,122,123]. The lamina cribrosa leads to an elongation and thinning of the lamina cribrosa, the volume of which may remain unchanged during axial elongation. The thinning of the lamina cribrosa in highly myopic eyes leads to a decrease in the distance between the intraocular compartment with the so-called IOP and the retrobulbar compartment with orbital cerebrospinal fluid pressure. The difference between both pressure parameters forms the trans-lamina cribrosa pressure difference. With the reduction in the distance between both compartment, the trans-lamina cribrosa pressure gradient, in the presence of an unchanged trans-lamina cribrosa pressure difference, steepens. This may be one of the reasons for an increase in the prevalence of optic nerve damage with a longer axial length in highly myopic eyes. Other intrapapillary reasons for an optic nerve damage may be the anatomic changes within the lamina cribrosa with a change in the arrangement of the lamina cribrosa pores through which the nerve fibers pass.

#### 3.9.3. Peripapillary Border Tissues

The intrapapillary compartment of the optic nerve head canal is separated from the surrounding tissue by border tissues [84,98]. The collagenous fibers of the peripapillary border tissue of the peripapillary scleral flange crisscross in a perpendicular angle with the collagenous fivers of the scleral flange, which continue into the lamina cribrosa [84,98,124]. They may be of importance for the stabilization of the optic nerve head structures in the sagittal direction, in particular for the stress and strain exerted on the lamina cribrosa by the ocular pulse. The peripapillary border tissue of the peripapillary scleral flange is the continuation of the optic nerve pia mater and demarcates the end of the lamina cribrosa. The peripapillary border tissue of the choroid separates the choroidal space from the intrapapillary compartment, is the continuation of the peripapillary border tissue of the peripapillary scleral flange, and ends at the end of BM. It thus connects the inner globe of the eye (including the uvea tract, BM, RPE, retina, lens, and vitreous body) with the outer shell, i.e., the sclera. Ocular eye movements are associated with marked acceleration and deceleration. Owing to its mass inertia, the inner globe will stay behind and show an aftermovement in relationship to the scleral shell, at which the external ocular muscles insert, and which primarily executes the eye movements. The shearing forces developing as a result of the reciprocal movements of the outer shell versus the inner globe will become effective at the structures connecting the inner globe with the outer shell. These structures are the scleral spur anteriorly and the peripapillary choroidal border tissue posteriorly. The latter will thus have biomechanical importance for the physiology of the eye.

The development of the gamma zone is associated with an increased distance between the end of BM and the optic disc border, i.e., an elongation and thinning of the choroidal peripapillary border tissue, the volume of which remains unchanged during axial elongation [90,91]. The biomechanical consequence of the gamma-zone-related elongation and thinning of the peripapillary choroidal border tissue has remained unclear so far. If the choroidal border tissue ruptures, the end of BM may become loose, leading to a corrugation of BM in that region.

#### 3.9.4. Parapapillary Alpha, Beta, Gamma, and Delta Zones

The parapapillary region contains four zones: the alpha zone, beta zone, gamma zone, and delta zone [84,103,125,126,127,128,129,130].

The alpha zone is characterized by the presence of an irregularly structured RPE on BM and is present in almost eyes, usually in the temporal region. The beta zone is characterized by the presence of BM and absence of the RPE, i.e., BM is uncovered or denuded of the RPE. It can be differentiated into a glaucomatous beta zone (potentially developed as a result of an IOP-fluctuation-related up rolling and unrolling of the RPE with eventual damage) and a myopic beta zone [131]. The latter is an enlargement of the RPE opening of the optic nerve canal, potentially without a loss of RPE cells. The RPE opening may enlarge as a result of increased strain within the RPE layer, caused by the enlargement of the inner BM surface in myopic eyes. The beta zone, independently of its etiology, is located centrally to the alpha zone and peripherally to the gamma zone, if present.

The gamma zone is located between the beta zone (or the alpha zone in the case of an absence of a beta zone) on its peripheral side and the peripapillary ring on its central side. It is defined by the absence of BM. The gamma zone develops in moderately myopic eyes as a result of a shift in the BM opening, usually in the foveal direction (i.e., temporal to temporal-inferior), as discussed above. A potential cause for the BM opening shift may be an enlargement (growth) of BM in the fundus midperiphery, peripheral to the optic nerve head. In highly myopic eyes, the BM opening enlarges (similar to, but not strictly parallel to, the enlargement of the RPE layer and of the lamina cribrosa), so that the nasally overhanging part of BM is retracted and a circular gamma zone, also present then in the nasal parapapillary, develops. In the gamma zone, the scleral inner surface has direct contact with the overlying retinal nerve fibers, covered by the inner limiting membrane. As BM with the RPE is as a complex that is not very strongly adherent with the overlying photoreceptors, the temporal movement of the end of BM may be more marked than the corresponding movement of the deep and middle retinal layers. This may explain the observation that some eyes with a gamma zone show an overhanging of the middle retinal layer and deep retinal layer into the peripheral region of the gamma zone. Owing to the absence of the RPE, in association with the almost complete absence of photoreceptors, the gamma zone represents an absolute scotoma.

The delta zone is defined as the region located within the gamma zone consisting of the area of an elongated and thinned peripapillary scleral flange. The latter is the continuation of the posterior sclera and extends into the lamina cribrosa. The border between the scleral flange and the lamina cribrosa is marked by the peripapillary border tissue of the scleral flange. The scleral flange is the anterior cover of the orbital cerebrospinal fluid space. In the delta zone, the inner surface of the scleral flange is covered by retinal nerve fibers and may contain occasionally large choroidal vessels, passing from the peripapillary arterial circle of Zinn–Haller to the lamina cribrosa. The thickness of the peripapillary scleral flange is positively correlated with the central lamina cribrosa thickness.

#### 3.9.5. Peripapillary Arterial Circle of Zinn–Haller

The peripapillary arterial circle of Zinn–Haller is located at the peripheral end of the scleral flange and nourishes the lamina cribrosa [132,133]. In eyes with a delta zone, the distance between arterial circle and the lamina cribrosa is enlarged as a result of the elongation of the peripapillary scleral flange. It may be of importance for an increased prevalence of optic nerve damage in highly myopic eyes.

#### 3.9.6. Suprachoroidal Parapapillary Cavitation

Suprachoroidal parapapillary cavitation (also called intrachoroidal parapapillary cavitation) is a cleavage between the choroid, connected to BM, and the sclera in the parapapillary region [134,135,136,137]. Usually positioned at the optic disc border in the temporal inferior or inferior parapapillary region, it is characterized ophthalmoscopically by an orange-like color. While its etiology has not been fully explored yet, it has been discussed that a backward pull by an optic nerve becoming too short to allow a full adduction of markedly axially elongated eyes may be responsible for the detachment of the sclera from the underlying choroid [138,139,140].

#### 3.9.7. Myopic Maculopathy (Myopic Macular Degeneration)

Myopic macular degeneration is differentiated into stage 1 (fundus tessellation), stage 2 (diffuse chorioretinal atrophy), stage 3 (extrafoveal patchy atrophies), and stage 4 (foveally located patchy atrophies) [83]. Fundus tessellation strongly correlated with a choroidal thinning at the posterior pole, thus marked fundus tessellation is a surrogate for a leptochoroid. Diffuse chorioretinal atrophy may not really be an atrophy, but just a further thinning of the posterior choroid, without a loss of tissue in the RPE layer and retina. Patchy atrophies are defects in the RPE layer in the macular region. While the pathogenesis of patchy atrophies remains unclear, it has been discussed that the axial-elongation-associated increase in the inner surface of BM leads to a stretching of the RPE layer, primarily causing an enlargement of the RPE opening of the optic nerve head (leading to the myopic parapapillary beta zone) and causing, in a second step, new defects in the RPE layer in the macular region. These macular RPE defects often surround a BM defect in its center.

Positive signs of myopic maculopathy are lacquer cracks and myopic neovascularization. Lacquer cracks may be linear defects in the RPE layer, eventually widening to areal patchy atrophies [95,141,142]. The orientation of the lacquer cracks is often perpendicular to the widest extension of the gamma zone. This supports the notion of an increased strain in the RPE layer, eventually leading to a relaxing linear defect at a perpendicular angle to the axis of the strongest strain.

Myopic maculoschisis is another feature of myopic traction maculopathy. As discussed above, its etiology may include an intraretinal shearing process, due to the backward slipping of BM away from the optic disc border (due to the development of the gamma zone) and the presumably firm adhesion of the inner limiting membrane at the optic disc border. Such a shearing process will be more marked in the region of a posterior staphyloma, which clinically may be a risk factor for maculoschisis.

Choroidal neovascularization as another characteristic of myopic maculopathy can eventually lead to an RPE cell proliferation into the subretinal space with the formation of a pseudo-fibrous metaplasia.

#### 3.9.8. Foveal Elevations in High Myopia

Elevations in the foveal region of highly myopic eyes can be differentiated into dome-shaped maculas and macular ridges [37,143,144,145,146,147,148,149]. A dome-shaped macula is a circular inward protrusion of the macula and is associated with a higher prevalence of macular BM defects and a relative thickening and relative inward protrusion of the central sclera and choroid. The spatial correlation with BM defects suggests a biomechanical role that may be played by BM in the development of a dome-shaped macula.

Macular ridges are fold-like elevations of the macula that can be detected in the OCT scan running at a perpendicular angle to the orientation of the fold. It has been discussed that macular ridges or folds are due to an asymmetrical enlargement of BM in the fundus midperiphery. To cite an example, if the enlargement of BM in the superior and inferior regions is larger than in the horizontal and nasal region, the surplus of BM at the posterior pole will lead to a horizontal fold in BM in the macular region.

#### 3.9.9. Myopia-Associated Optic Neuropathy

High myopia may be a risk factor for non-glaucomatous, glaucoma-like, and glaucomatous optic nerve damage [88,150,151,152,153]. The non-glaucomatous optic neuropathy may potentially be explained by a stretching of the retinal nerve fibers, caused by the increased distance between the retinal ganglion cell axons and the optic disc. The glaucoma-like optic nerve damage may be defined by a loss in neuroretinal rim and an abnormal shape of the neuroretinal rim, in particular, notches in the rim in the inferior and superior optic disc regions. As these changes often occur in eyes with a statistically normal IOP and because a study showing the therapeutic benefit of an IOP reduction has not been available so far, it has remained unclear whether this type of optic neuropathy in highly myopic eyes is glaucomatous or just glaucoma-like. A glaucomatous optic nerve damage occurs in highly myopic eyes with an elevated IOP and a typical glaucomatous appearance of the optic nerve head (namely, a loss in neuroretinal rim area and an abnormal shape of the neuroretinal rim). Risk factors for a glaucoma-like and glaucomatous optic neuropathy may be an enlargement of the lamina cribrosa in association with its elongation and thinning, an elongation and thinning of the peripapillary scleral flange (parapapillary delta zone) as the biomechanical anchor of the lamina cribrosa, and the increased distance between the peripapillary arterial circle of Zinn–Haller and the optic disc due to the elongation of the peripapillary scleral flange. 

#### 3.9.10. Definition of High Myopia

While a definition of high myopia has not generally been agreed upon, high myopia may be defined, from a morphological point of view, by an enlargement of the BM opening [99,154,155,156]. Studies suggested that, at an axial length of 26.0 mm to 26.5 mm, the probability of an enlargement of BM opening markedly increases, such that the value may be discussed to roughly represent a border between moderate myopia and high myopia.

## 4. Discussion

**Figure 1:**
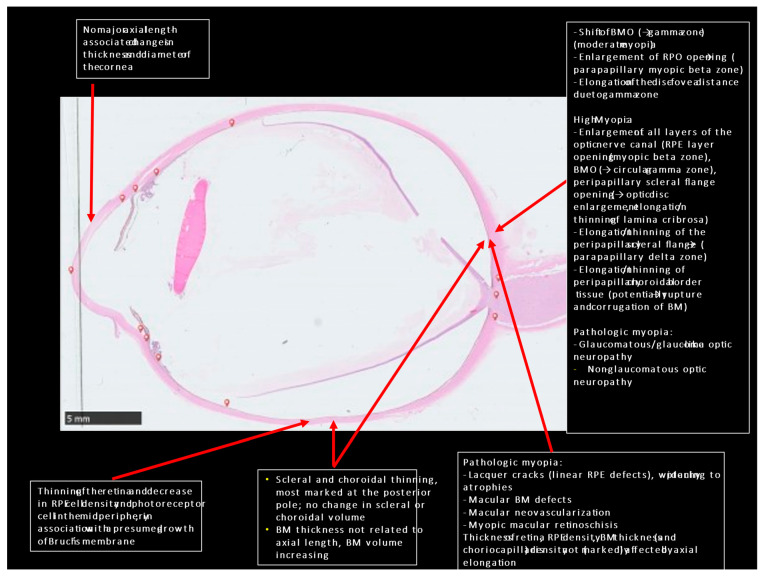
Histo-photograph of a highly myopic eye with an artificially detached retina, and lists of morphological features of high myopia and pathological myopia.

Combining the anatomical findings obtained in histological and clinical studies may lead to the hypothesis that myopic axial elongation is achieved primarily by an enlargement (perhaps active growth) of BM in the midperiphery of the fundus [57] (Figure 1). Such a mechanism could explain the thinning of the choroid at the posterior, a finding that cannot be explained when the sclera is considered to be the primary driver of eye elongation. A primary scleral backward movement would lead to an increased distance between the scleral inner surface and BM, i.e., a thickening of choroid. A primary BM enlargement in the fundus midperiphery would also explain the change in eye shape from a sphere to a prolate ellipsoid, choroidal thinning most pronounced at the posterior pole, retinal thinning and RPE cell density reduction in the fundus midperiphery, unchanged retinal thickness, RPE cell density and choriocapillaris thickness at the posterior pole, temporal shift of the BM opening with the development of a gamma zone, elongation of the optic disc–fovea distance and decrease in angle kappa, and a BM thickness not related to axial length. The development of a parapapillary myopic beta zone, the enlargement of the BM opening, and the development of macular defects in the RPE layer and eventually in BM may be due to an increased strain in the RPE layer and BM due to the increase in the coronal eye diameters. Macular ridges (folds) may be explained by an asymmetry of BM enlargement in the fundus midperiphery. The presumed location of the fundus midperiphery as the main location of an enlargement of BM fits with the results of experimental and clinical studies suggesting that the fundus midperiphery is the location of the afferent part of the intraocular feedback mechanism of the regulation of axial elongation in the process of emmetropization and myopization.

## 5. Practical Implications

Future studies may explore the messenger molecule potentially produced in the retina in the fundus midperiphery and the possibility of causing the RPE at the same location to produce more and new BM, resulting in a BM enlargement.

## 6. Study Limitations

The volume of the topic did not allow a complete description of all morphological changes found in association with myopic axial elongation, and the discussed possibility of a BM enlargement in the fundus midperiphery as a main element in axial elongation will be only one out of several possibilities to try to explain the process of axial elongation. Not mentioning other hypotheses in this review, in particular those focusing on the potential role the choroid and sclera may play in axial elongation, does not mean that these hypotheses are not valid.

## Data Availability

The data are available from the corresponding author upon reasonable request.
